# Supplementary material for: Polymorphisms in Genes Encoding VDR, CALCR and Antioxidant Enzymes as Predictors of Bone Tissue Condition in Young, Healthy Men
Source: Int J Mol Sci. 2023 Feb 8;24(4):3373. doi: 10.3390/ijms24043373 (PMC9964706; doi:10.3390/ijms24043373)
Supplement: Supplementary file 1 [file ijms-24-03373-s001.zip › ijms-2161822-supplementary.pdf]

A

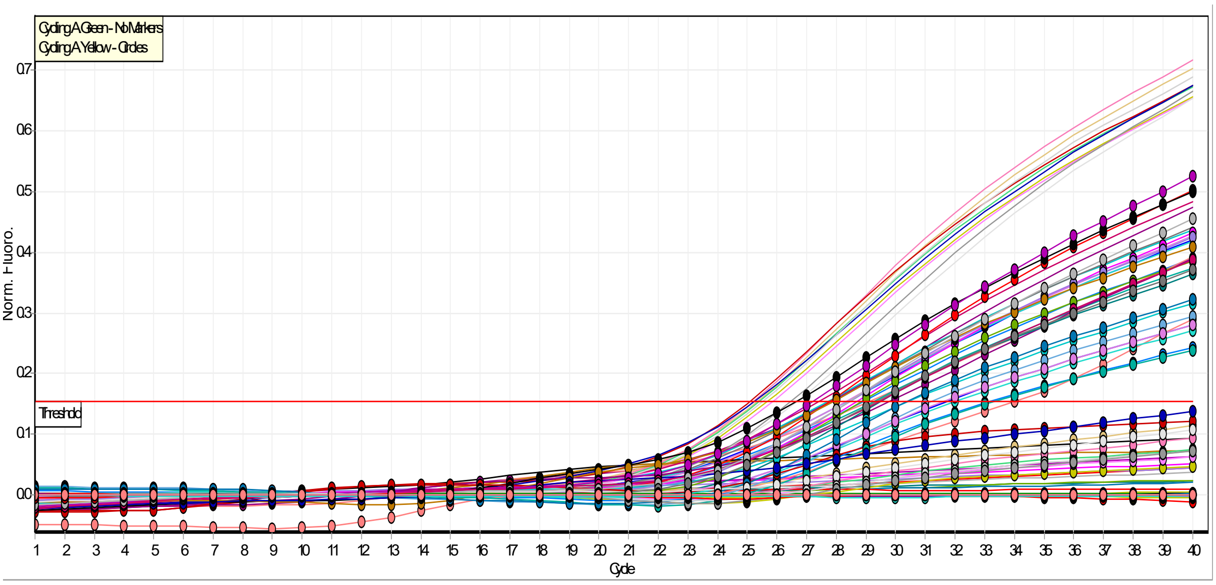
Cycling A.Green - No Markers
Cycling A.Yellow - Circles
Norm. Fluoro.
Threshold
Cycle

B

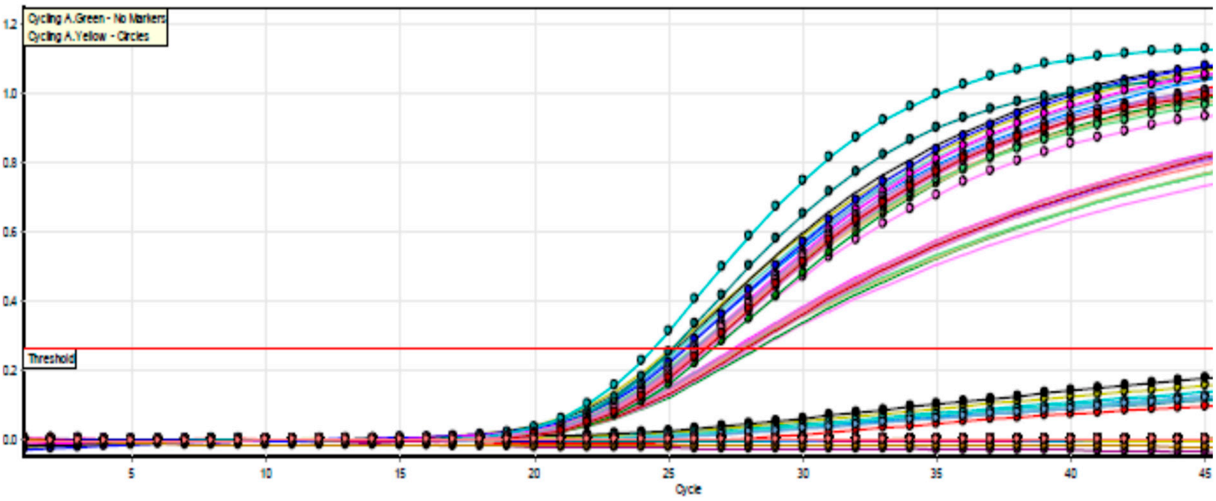
Cycling A.Green - No Markers
Cycling A.Yellow - Circles
Threshold
Cycle

C

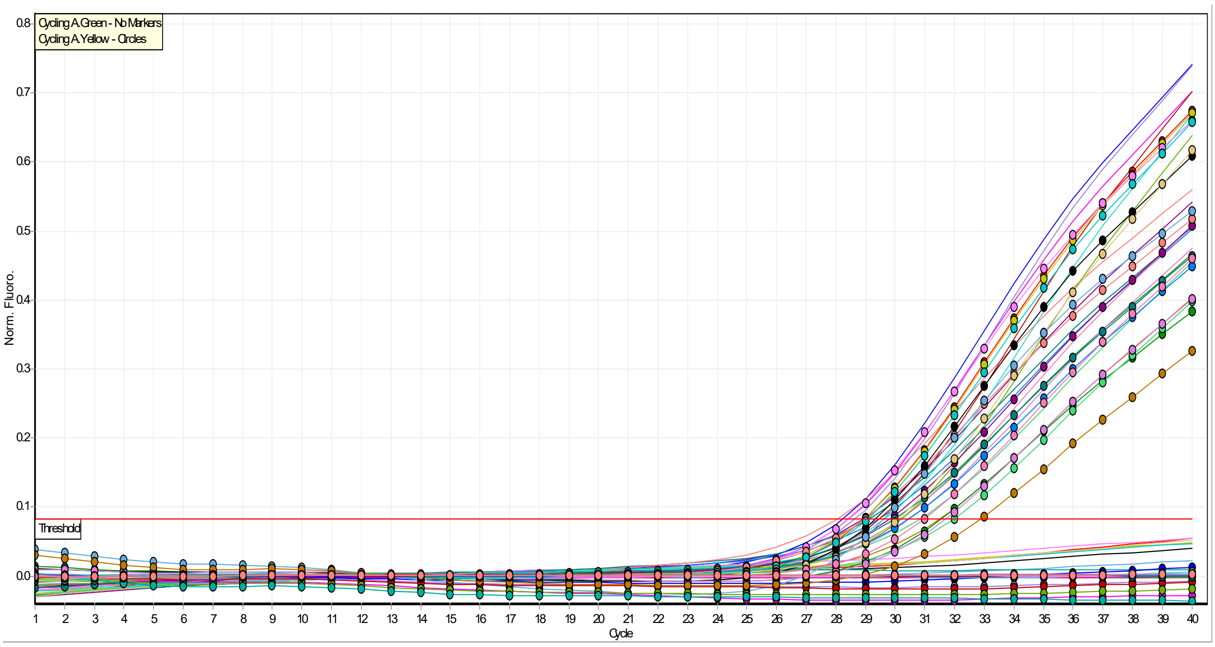
Cycling A.Green - No Markers
Cycling A.Yellow - Circles
Threshold
Norm. Fluoro.
Cycle
0.8
0.7
0.6
0.5
0.4
0.3
0.2
0.1
0.0
1 2 3 4 5 6 7 8 9 10 11 12 13 14 15 16 17 18 19 20 21 22 23 24 25 26 27 28 29 30 31 32 33 34 35 36 37 38 39 40

D

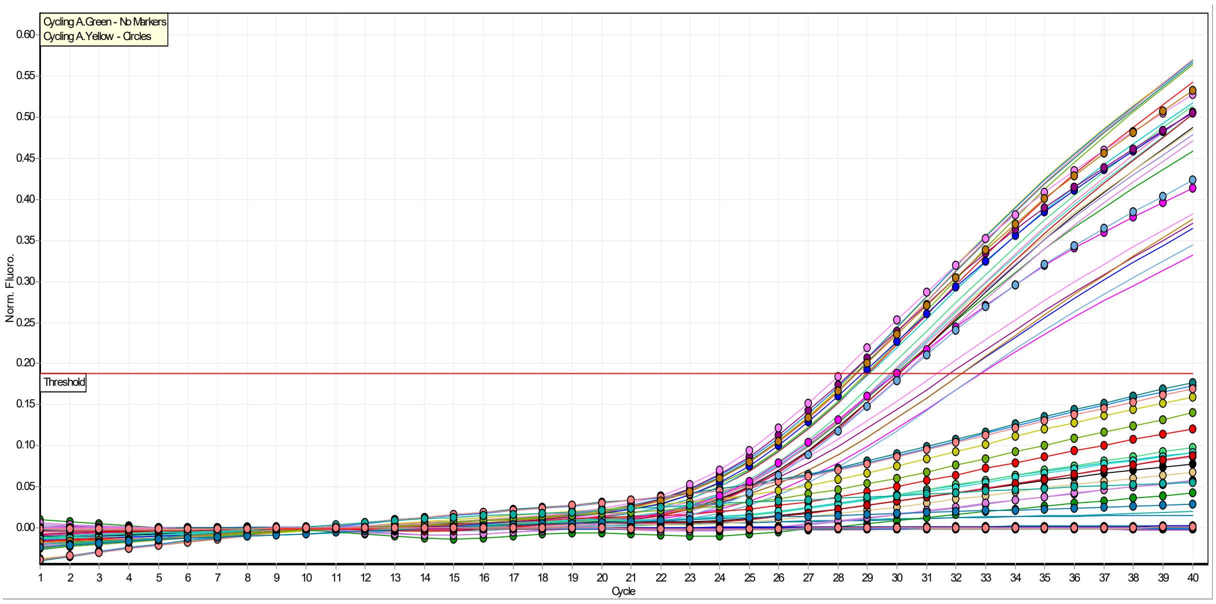
Cycling A.Green - No Markers
Cycling A.Yellow - Circles
Threshold
Norm. Fluoro.
Cycle
0.60
0.55
0.50
0.45
0.40
0.35
0.30
0.25
0.20
0.15
0.10
0.05
0.00
1 2 3 4 5 6 7 8 9 10 11 12 13 14 15 16 17 18 19 20 21 22 23 24 25 26 27 28 29 30 31 32 33 34 35 36 37 38 39 40

E

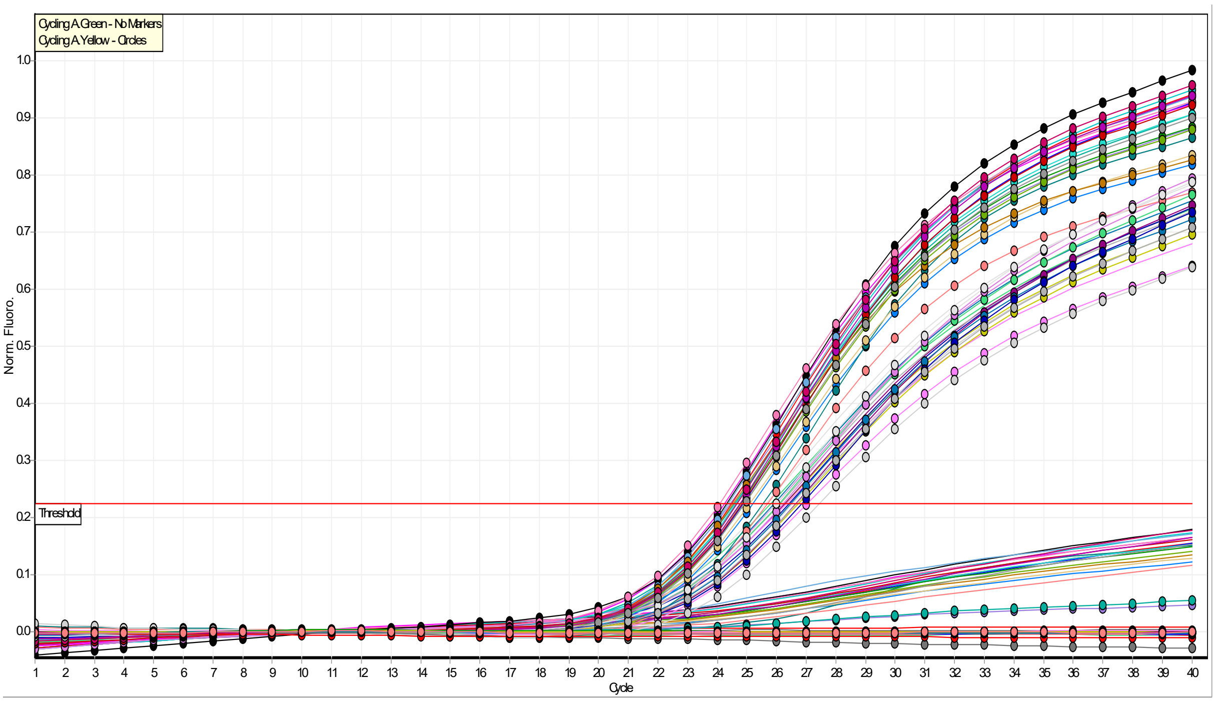
Cycling A.Green - No Markers
Cycling A.Yellow - Circles
Threshold
Norm. Fluoro.
Cycle

F

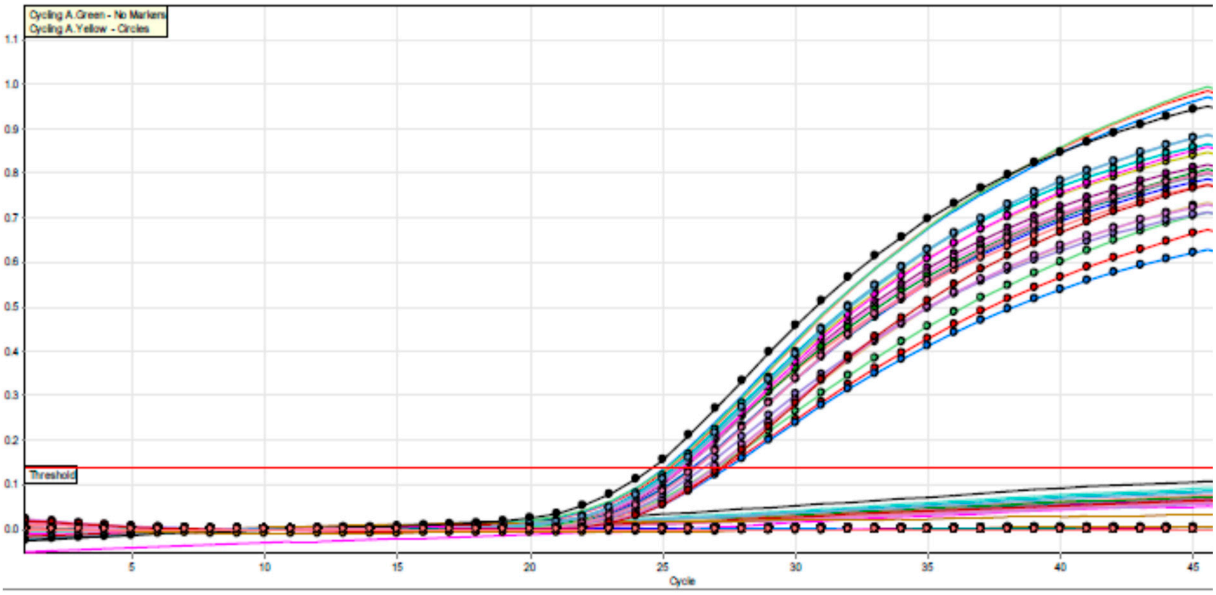
Cycling A.Green - No Markers
Cycling A.Yellow - Circles
Threshold
Cycle

G

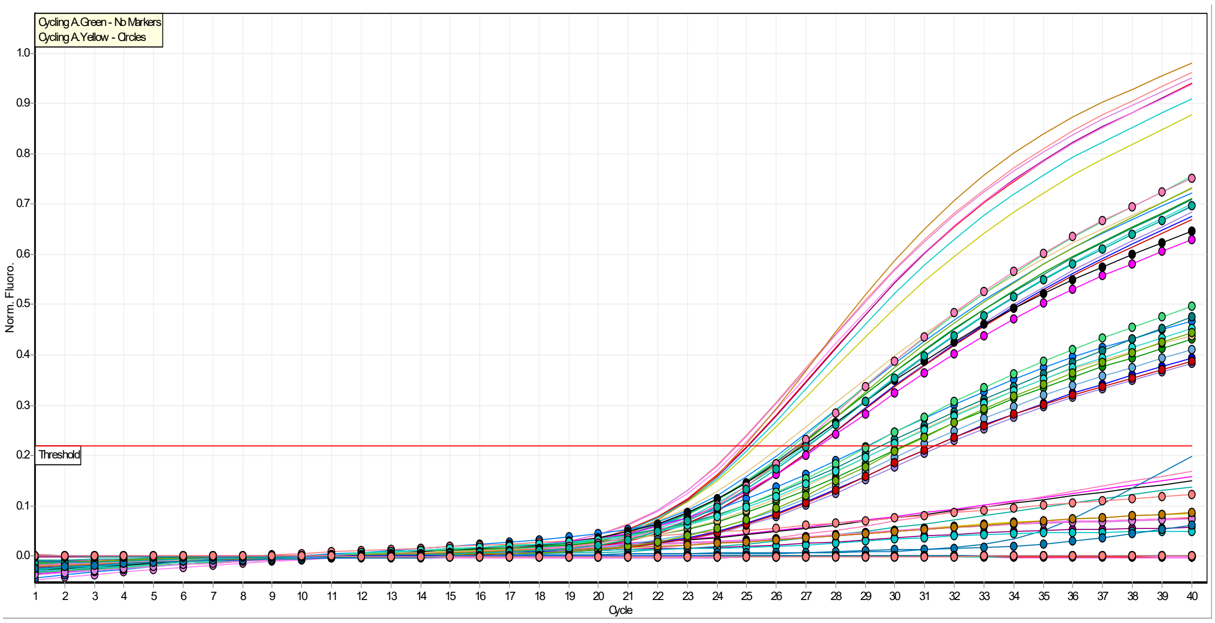

H

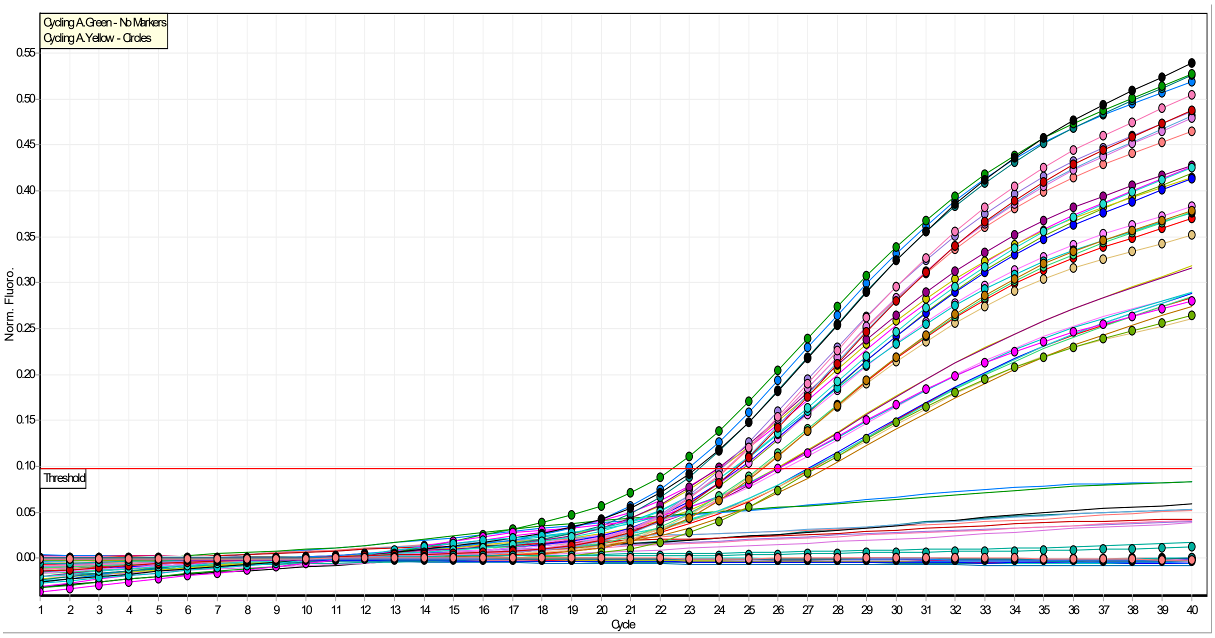

Figure S1. Sample photos of the TaqMan analyzes results for each of the tested polymorphisms.

A- *VDR ApaI*; B- *VDR BsmI*; C- *VDR FokI*; D- *COL1A1*; E- *CALCR*; F- *SOD1*; G- *SOD2*; H- *GPx*
